# When Neisseria meningitidis Strikes an Immunocompetent Host and Purpura Fulminans Results

**DOI:** 10.7759/cureus.84350

**Published:** 2025-05-18

**Authors:** Andy Li, Parima Saxena, Aye M Thida, Ekenedilichukwu N Nnadi, Jordonna Brown

**Affiliations:** 1 Internal Medicine, State University of New York Downstate Medical Center, Brooklyn, USA; 2 Hematology and Medical Oncology, State University of New York Downstate Medical Center, Brooklyn, USA

**Keywords:** disseminated intravascular coagulation (dic), fulminans purpura, immuno-competent host, neisseria meningitidis, organ failure from sepsis

## Abstract

Purpura fulminans (PF) is a rapidly progressive, life-threatening condition marked by ecchymotic, tender, and symmetric skin lesions, often resulting from disseminated intravascular coagulation (DIC) secondary to infectious or non-infectious etiologies. PF usually occurs in immunocompromised hosts and is associated with a high mortality rate. Here, we highlight a case of an elderly immunocompetent woman with *Neisseria meningitis-mediated* DIC and subsequent PF. Her case underscores the importance of early intervention in managing this rare but deadly condition in order to ensure optimal survival outcomes. Early intervention for PF with DIC includes the timely administration of antibiotics, steroids, and anticoagulants, along with addressing the underlying consumptive coagulopathy.

## Introduction

Purpura fulminans (PF) is a rapidly progressive, life-threatening disorder classified by the new appearance of ecchymotic, tender, and symmetric skin lesions involving the limbs and is thought to be the sequelae of both infectious and non-infectious etiologies [1]. It can be seen as a rare manifestation of disseminated intravascular coagulopathy (DIC), occurring due to diffuse intravascular thrombosis and hemorrhagic infarction of the skin. While PF can also be triggered by various micro-organisms, one of the most concerning culprits is *Neisseria meningitidis*, known to cause meningococcal infection. Although PF associated with sepsis from *Neisseria meningitidis* is exceedingly rare in older adults, it remains a dreaded cause due to substantial morbidity and mortality [2]. Multiple cases in the literature have highlighted the role of *Neisseria *meningitidis in sepsis often leading to amputation and tissue loss, often in young and immunocompromised patients [3-5]. The incidence of PF is more common amongst children and young adults, and is more limited in middle-aged and elderly patients [5]. Here, we present a unique case of PF secondary to DIC caused by *Neisseria meningitidis* in an immunocompetent elderly patient, highlighting the importance of early recognition and timely management of this life-threatening condition.

## Case presentation

**Figure 1:**
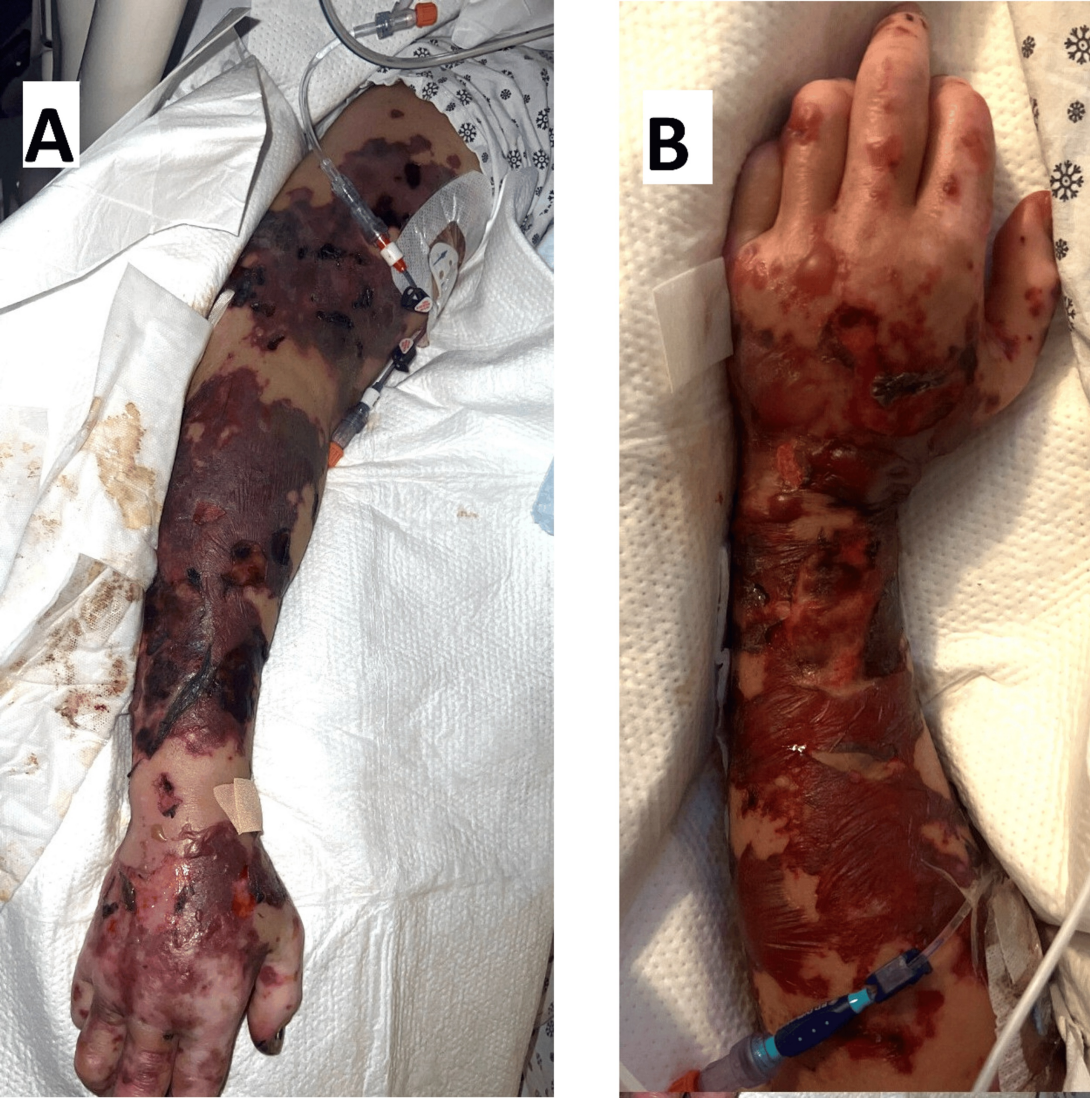
Purpura fulminans rash throughout admission. Panel A shows rash on presentation. Panel B shows progression of rash throughout ICU course.

A 68-year-old female with a past medical history of migraine disorder, gastritis, and obesity was brought to the emergency department with altered mental status after being found unresponsive at home. On initial evaluation, the patient’s vital signs were notable for normal blood pressure of 104/82 mmHg, tachypnea respiratory rate of 24 breaths per minute, mild tachycardia heart rate of 105 beats per minute, and hypothermia, temperature of 96.3 F. Physical examination was significant for extensive scattered purpuric patches observed throughout the body and tender to palpation (Figure 1A). She also had a Glasgow Coma Scale score of 13. Physical examination was negative for nuchal rigidity, Kernig sign, and Brudzinski sign. The family at the bedside denied any sick contact, and her vaccination history was unknown. She had no prior history of altered mental status, recent hospitalizations, HIV, malignancy or autoimmune disease.

Initial laboratory tests revealed hemoglobin 10.2 g/dL, a normal white blood cell count of 5.65, platelet count of 83,000, elevated D-dimer level of 31,984, and an elevated lactic acid level of 4.1 mmol/L (Table 1). All other laboratory values were within normal limits. A peripheral smear showed schistocytes, predominantly granulocytosis, including toxic neutrophils. Chest and abdominal imaging were benign, and ultrasound of bilateral lower extremities was negative for deep vein thrombosis. Blood cultures were taken and followed immediately by empiric broad-spectrum antimicrobials (vancomycin, cefepime, and metronidazole). Subsequently, the patient was admitted to the intensive care unit (ICU) with a diagnosis of septic shock of unknown etiology requiring vasopressor support. 

**Table 1:** Summary of laboratory values

Test	Result	Reference Range
Hemoglobin	10.2 g/dL	12 - 16 g/dL
White Blood Cell Count	5.65 K/uL	3.80 - 10.80 K/uL
Platelet Count	83 K/uL	150 - 400 K/uL
D-dimer	31,984 ng/mL	<=340 ng/mL
Lactic Acid	4.1 mmol/L	0.5 - 1.6 mmol/L
Activated Partial Thromboplastin Time (aPTT)	45 seconds	25 - 37 seconds
Prothrombin Time (PT)	20.0 seconds	9.4 - 12.5 seconds
Platelet Count (ICU day 7)	56 K/uL	130 - 400 K/uL
International Normalized Ratio (INR)	1.8	0.8 - 1.2 ratio
Haptoglobin (ICU day 7)	<20 mg/dL	50 - 220 mg/dL
Lactic Acid Dehydrogenase (LDH) (ICU day 7)	633 U/L	135 - 214 U/L
D-dimer (ICU day 7)	46,844 ng/mL	<=340 ng/mL

Our patient’s ICU course was complicated by DIC characterized by prolonged activated partial thromboplastin time (aPTT) (45 seconds), prolonged prothrombin time (PT) (20.0 seconds), thrombocytopenia (56 K/uL), elevated international normalized ratio (INR) (1.8), low haptoglobin (<20 mg/dL), elevated lactic acid dehydrogenase (633 U/L), and elevated D-dimer (46,844 (Table 1).

She was started on pulse dose steroids of hydrocortisone and fresh frozen plasma (FFP), as protein C concentrate was unavailable. She was started on therapeutic dose anticoagulation with heparin infusion.

Blood cultures came back positive for *Neisseria meningitidis*, confirming the diagnosis of meningococcemia with purpura fulminans. Subsequently, the patient’s antibiotic regimen was narrowed to ceftriaxone 2 g IV twice daily. Furthermore, pathological skin examinations via skin biopsy of purpuric lesions cemented the diagnosis of purpura fulminans. Her ICU course continued to be complicated by multiple organ failure such as acute hypoxic respiratory failure requiring intubation, renal failure resulting in need for dialysis, encephalopathy, and worsening skin changes with extensive bullae (Figure 1B).

Her condition gradually improved over the span of two weeks in the ICU with recovery of her organ functions. She was eventually weaned off mechanical ventilation and extubated. The patient's clinical status continued to improve, and she was later discharged to subacute rehab.

## Discussion

Here, we highlight a unique case of* Neisseria meningitidis *sepsis progressing to DIC and PF. A review of our patient’s hospital course highlights the timely nature of early recognition and broad-based clinical management to facilitate a life-saving outcome. 

Meningococcal disease remains a feared etiology of bacterial sepsis caused by *Neisseria meningitidis*. It has the highest incidence in infants less than one year of age, followed by the 17-21 age group, and lastly, an increased incidence in adults older than 85 [5]. Immunocompromised individuals also have an increased risk of contracting *Neisseria meningitidis* [3-6]. Despite this, our patient presented with meningococcemia in her six decades of life without a history of immunocompromisation or recent exposure to sick contacts. Classic manifestations of meningococcal disease include fever, altered mental status, and nuchal rigidity. A cohort study showed that the presence of a rash was highly associated with at least one other manifestation noted above, with 89% of patients having two or more of these clinical findings [7]. The rash associated with *Neisseria meningitidis* usually presents as petechiae or a blanching purpura rash that may progress to non-blanching, correlated with severe disease [8]. However, our patient presented with DIC, leading to PF, which is only seen in 10-20% of patients with meningococcal sepsis [9].

PF is a rare but fatal complication of DIC, with a mortality rate of over 50% [10]. The pathophysiology involves the dysregulation of the coagulation cascade, causing a hypercoagulable state that leads to occlusion of the blood vessels of the skin [10]. PF is generally associated with three different etiologies: hereditary, post-infectious/idiopathic, and acute infection. The hereditary form presents early on in neonates and is associated with the loss of anticoagulant proteins such as Protein C, Protein S, and antithrombin III. The post-infectious etiology is likely secondary to autoimmunity and is usually seen 7-10 days after an acute infection, although anti-Protein C and Protein S antibodies are rarely detected [10]. The acute infectious/sepsis-induced cause of PF was seen in our patient, whose skin lesions rapidly developed in the setting of DIC and septic shock. While DIC is typically associated with a depletion of clotting factors, the presence of PF further complicates clinical management as it becomes necessary to strike a balance between the rapid consumption of clotting factors and hypercoagulability.

The treatment of DIC is supportive, with the goal of preventing hypercoagulability and consumption of coagulation factors. Supportive care can involve correcting the underlying etiology and administering vitamin K, cryoprecipitate, and Protein C or FFP. The role of anticoagulation in DIC remains controversial; however, in the case of widespread and clinically noticeable thrombosis such as purpura fulminans, it becomes imperative to begin an anticoagulation regimen such as heparin [11]. Careful monitoring of laboratory values becomes essential in order to maintain the delicate balance between a disease process that activates both pro- and anti-coagulative factors. In this patient, we titrated the heparin dosage against factor anti-Xa as the measurement of partial thrombin time (PTT) would be nonspecific, given the cascade consumption by DIC. 

Despite the atypical demographic for *Neisseria meningitidis*, further compounded by a rare complication, infectious PF, rapid recognition of the disease, and starting immediate treatment was critical to this patient’s successful outcome. Although a skin biopsy is the gold standard for confirmation of purpura fulminans, the high mortality rate due to widespread organ failure urgently demands initiating therapy [10]. By targeting the infectious cause, meningococcal sepsis, with appropriate antibiotics, administering steroids to prevent Waterhouse-Freiderichsen syndrome, and providing both heparin and Protein C supplementation, the patient made a full recovery from an otherwise highly fatal condition.

While our case highlights the success of early recognition of PF, we may have had a more robust outcome by considering this diagnosis at admission. This may have prevented septic shock in our patient. Our index of suspicion at onset was limited by the limited literature demonstrating the feared consequences of PF in immunocompetent patients. 

## Conclusions

Infectious purpura fulminans is a hematological emergency that requires quick recognition and prompt treatment to reduce the risk of severe complications and death. A distinct skin rash is a key diagnostic indicator and should trigger immediate therapy, as waiting for biopsy results could delay diagnosis and worsen outcomes. In cases of acute infectious purpura fulminans, anticoagulation is considered if DIC is present. Along with comprehensive supportive care and urgent broad-spectrum antibiotics, PF with DIC necessitates addressing consumption coagulopathy and prevention of hypercoagulability.
